# A self-stiffening compliant intracortical microprobe

**DOI:** 10.1007/s10544-024-00700-7

**Published:** 2024-02-12

**Authors:** Naser Sharafkhani, John M. Long, Scott D. Adams, Abbas Z. Kouzani

**Affiliations:** School of Engineering, Deakin University, Geelong, VIC 3216 Australia

**Keywords:** Intracortical microprobe, Insertion, Buckling, Self-stiffening, Finite element method, 3D printing

## Abstract

Utilising a flexible intracortical microprobe to record/stimulate neurons minimises the incompatibility between the implanted microprobe and the brain, reducing tissue damage due to the brain micromotion. Applying bio-dissolvable coating materials temporarily makes a flexible microprobe stiff to tolerate the penetration force during insertion. However, the inability to adjust the dissolving time after the microprobe contact with the cerebrospinal fluid may lead to inaccuracy in the microprobe positioning. Furthermore, since the dissolving process is irreversible, any subsequent positioning error cannot be corrected by re-stiffening the microprobe. The purpose of this study is to propose an intracortical microprobe that incorporates two compressible structures to make the microprobe both adaptive to the brain during operation and stiff during insertion. Applying a compressive force by an inserter compresses the two compressible structures completely, resulting in increasing the equivalent elastic modulus. Thus, instant switching between stiff and soft modes can be accomplished as many times as necessary to ensure high-accuracy positioning while causing minimal tissue damage. The equivalent elastic modulus of the microprobe during operation is ≈ 23 kPa, which is ≈ 42% less than the existing counterpart, resulting in ≈ 46% less maximum strain generated on the surrounding tissue under brain longitudinal motion. The self-stiffening microprobe and surrounding neural tissue are simulated during insertion and operation to confirm the efficiency of the design. Two-photon polymerisation technology is utilised to 3D print the proposed microprobe, which is experimentally validated and inserted into a lamb’s brain without buckling.

## Introduction

Neural microprobes contribute significantly to the study of brain function, brain diseases, and brain-machine interfaces (Li et al. 2023; McGlynn et al. 2022; Ward et al. 2022). An intracortical microprobe needs to be implanted deep within the brain to directly interface with specific brain regions allowing for high-accuracy brain recording and stimulation (Atkinson et al. 2021; Zhou et al. 2023). An inserter is utilised to push a microprobe into the brain to penetrate its surface and place the microprobe at a desired depth. The brain penetration force, which describes the resistance encountered by a microprobe during insertion, is determined by different factors, including characteristics of targeted tissue, a microprobe design, and speed of insertion (Schiavone et al. 2009; Sharp et al. 2009). A rat brain can be penetrated with a force of 0.5-2 mN after a dura mater has been removed (Arafat et al. 2019; Sharp et al. 2009). The dura mater is the outermost layer of three protective layers surrounding the brain.

Because of the large difference between the elastic moduli of neural tissue and an implanted fixed microprobe, brain micromotion, caused by physiological and behavioural movements, damages surrounding neural tissue during operation (Duncan et al. 2021; Sharafkhani et al. 2022a; Zhang et al. 2023). The tissue damage activates the brain’s immune system, which adversely affects the microprobe’s functionality, and may lead to its isolation and failure within weeks or months (Fiáth et al. 2019; Mohammed et al. 2020). Utilising flexible microprobes with elastic moduli close to that of the brain, ≈ 0.1–16 kPa (Hong et al. 2018), significantly mitigates tissue damage (Ferro et al. 2018; Yang et al. 2019). Nevertheless, insertion failure occurs as a result of a reduction in critical buckling force below the force required to penetrate the brain surface (Sharafkhani et al. 2022a).

To date, most studies have focused on temporarily stiffening flexible intracortical microprobes to prevent buckling during insertion using insertion shuttles or bio-dissolvable coatings (Pimenta et al. 2021; Sharafkhani et al. 2022b; Tang et al. 2020; Zátonyi et al. 2019; Zhang et al. 2020; Zhang et al. 2021). A 5 mm long polymer-based neural microprobe was proposed whose elastic modulus decreases from 2 GPa to 300 MPa within 10 min as a result of contact with physiological conditions (Zátonyi et al. 2019). Similarly, a fiber-shaped microprobe’s elastic modulus decreases from 10 GPa to 10 kPa in 25 min (Tang et al. 2020). During insertion, a polyimide-based microprobe was engaged with a steel shuttle by a PEG coating which dissolves 50 s after penetration, resulting in the microprobe releasing and the shuttle retracting (Zhang et al. 2020). However, the inability to adjust the dissolving time following the microprobe contact with cerebrospinal fluid is a big challenge. Moreover, it is impossible to regain the stiffness after it has been prematurely lost, which could adversely affect the positioning accuracy. Therefore, replacing the microprobe with a new one is the only solution to correct a positioning error which may cause further tissue damage. Although a piezoelectric-based mechanism, proposed to actively control the stiffness of a polyimide microprobe by applying voltage, addressed the mentioned challenges (Sharafkhani et al. 2022b), a new challenge arises in guaranteeing the accurate, effective, and comprehensive attachment of the piezoelectric layers to the surrounding structure. The seamless integration is crucial to withstand the stress applied to the interface during both insertion and operation and to keep functionality.

This study proposes a self-stiffening compliant intracortical microprobe, whose design has been optimised from one previously reported by the authors (Sharafkhani et al. 2023a; Sharafkhani et al. 2023b) to reduce the equivalent elastic modulus from ≈ 40 kPa to ≈ 23 kPa during operation. The previously reported design was composed of only one compressible structure at the microprobe base. In the current design, a second compressible structure has also been added, resulting in ≈ 46% less maximum strain generated under brain longitudinal motion, confirming its great compliance with surrounding tissue during operation. The equivalent elastic modulus increases to ≈ 4.2 GPa due to the axial compression applied by an inserter. Hence, instant switching between stiff and soft modes can be accomplished as many times as necessary to correct positioning errors and minimise tissue damage during accurate positioning. The microprobe and surrounding neural tissue are simulated during insertion and operation to confirm the efficiency of the optimised design.

A variety of 3D printing technologies, including fused deposition modeling (FDM), stereolithography (SLA), and selective laser sintering (SLS), can be utilised to manufacture highly customisable microcomponents for use in biomedical applications (Rogkas et al. 2022). Two-photon polymerisation (2PP) technology enables the creation of exceptionally high-resolution structures with fine details (Marschner et al. 2023). Furthermore, its capacity to achieve precision at a relatively fast rate enhances efficiency in a prototyping process. 2PP technology creates smooth surfaces, making the printed microstructures suitable for applications where surface quality is important, particularly for fabricating those that interact with biological tissue. Hence, considering the complexity of the design, 2PP technology is utlised to fabricate the proposed self-stiffening intracortical microprobe. Unlike the discussed piezoelectric-based mechanism, the proposed microprobe is constructed from a single material, thereby eliminating the corresponding challenge mentioned above. The 3D printed microprobe is experimentally validated and successfully inserted into a lamb brain to confirm its resistance against buckling.

## Materials and methods

### Design

**Fig. 1:**
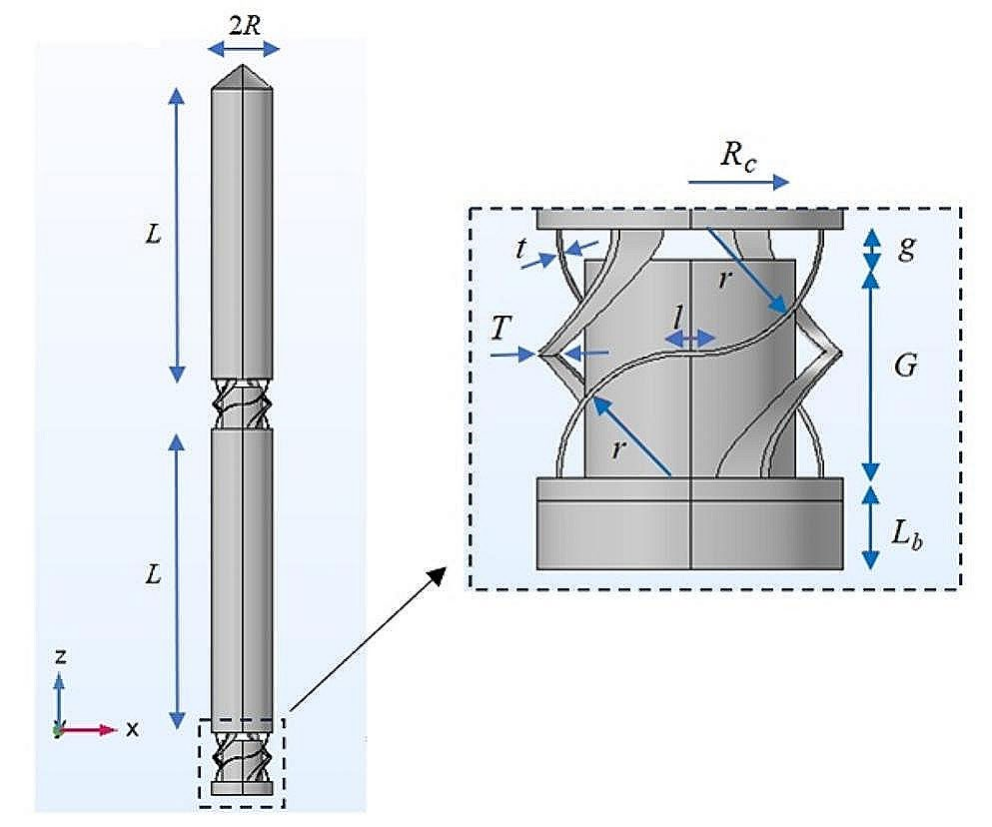
A side view of the proposed intracortical microprobe with two compressible structures

Figure 1 illustrates the proposed self-stiffening compliant intracortical microprobe composed of two *L* long cylinders with a radius *R* mounted on two compressible structures with the same radius. A tip section is placed on the top of the upper cylinder. The lower compressible structure is placed on a *L*_*b*_ long cylindrical base (a zoomed-in view of Fig. 1). The compressible structure comprises four *t* thick curved legs, whose width at the middle is *T*. A small cylinder with a radius of *R*_*c*_ = *R*-*T*-*q*, and a length of *G* is placed at a *g* distance from the upper part. As depicted in the zoomed-in view, every curved leg comprises two quarter-ring-like structures with radii of *r* (*r* > *g*) whose centres are located at vertical and lateral distances 2*r* + *t*, and *l*, respectively. Table 1 lists the geometrical properties of the proposed microprobe. Considering the microprobe’s specific design, *t* = 3 μm is the minimum thickness which enables fabrication.

**Table 1:** Geometrical properties of the proposed microprobe

Parameter	*L*	*r*	*T*	*g*	*G*	*L*_*b*_	*l*	*t*	*q*
Value (µm)	1000	85	10	30	137	20	10	3	20

During insertion, an inserter pushes the microprobe toward the brain surface by applying an axial force, *F*, to its base. Hence, the whole microprobe, except the upper cylinder and tip, starts moving in the + z direction due to contracting the compressible structures. By increasing the applied force and reaching the generated displacement of *w* = 2*g* for the base, the gaps in the compressible structures are filled, and the microprobe converts from a compressible structure (soft mode) to an integrated semi-cylindrical one (stiff mode). In other words, the proposed microprobe is soft for *w* < 2*g* and is stiff for *w* ≥ 2*g* whose axial stiffness could be calculated as (Hibbeler 2017),

1 $${K}_{i}=\frac{\text{d}F}{\text{d}w}$$

in which “*i”* stands for “soft” (*w* < 2*g*) and “stiff” (*w* ≥ 2*g*). Assuming the microprobe as a classic cylindrical structure with a length of *2L + 2g + 2G + L*_*b*_ and a radius of *R*, the equivalent elastic modulus, *E*_*i*_, can be obtained by (Hibbeler 2017),

2 $${E_i} = \eta \frac{{{K_i}(2L + 2G + 2g + {L_b})}}{{\pi {R^2}}}$$

where a correction factor, *η*, is employed to account for the irregular shape of the proposed microprobe.

During operation, the surrounding neural tissue’s micromotion applies an axial force to the microprobe, shifting its tip in the -z direction. If the created displacement, *w*, is less than 2*g*, the microprobe will be soft and act like a spring under brain physiological motion.

Considering the compressible structures’ design, the following relationship is defined to let the structure contract with the minimum possible radius, *R*.

3 $$R=0.8\left(2r+l\right)/\sqrt{2}$$

Utilising focused ion beam scanning electron microscopy (FIB-SEM) enables the precise deposition of platinum patterns onto the cylinders and the compressible sections’ curved legs to create conductive traces and recording sites.

### Simulation

COMSOL Multiphysics 5.6 (“Solid Mechanics” module - “Stationary” study) is employed to simulate the proposed intracortical microprobe during insertion and operation. The obtained results are compared with those of the microprobe with one compressible structure (CS) (Sharafkhani et al. 2023b) to confirm the efficiency of the proposed design. The radius and length of the simulated two microprobes are the same. The modelled microprobes are made of a biocompatible resin, IP-S, with an elastic modulus of 4.2 GPa, a density of 1300 kg/m^3^, and a Poisson’s ratio of 0.3 (Kramer et al. 2020). The neural tissue is modelled as a “block” with cross-sectional dimensions of 2(*R* + 750) µm×2(*R* + 750) µm and a height of 2*L* + *G* + *g* + 750 μm. Assuming a linear elastic and isotropic model, the neural tissue is defined as a “Blank Material” with a density of 1042.50 kg/m^3^, an elastic modulus of 6 kPa, and a Poisson’s ratio of 0.45 (Mahajan et al. 2020; Nguyen et al. 2014).

As the microprobe is fixed to an inserter during insertion, the microprobe base’s movements in the x and y directions are restricted. Furthermore, to simulate the applied force by the inserter, the base is subjected to different compressive forces in the + z direction via the “Boundary Load” node. Before reaching the applied force to the brain penetration force, the microprobe tip does not move. Hence, the pinned boundary condition is applied to the simulated microprobe tip. The base displacement of the simulated microprobe against different applied forces is obtained to calculate the corresponding equivalent elastic moduli for the soft and stiff modes. Furthermore, the strain generated on the brain surface during insertion is calculated. Longitudinal, *W*, motion is applied to the bottom surface of the modelled neural tissue via the “Prescribed displacement” node to simulate the brain longitudinal motion during operation. The displacements of the microprobe and the maximum principal strain generated under brain motion are calculated. The average mesh quality during insertion and operation is 0.65 and the relative tolerance sets at 0.001.

### Fabrication

Photonic Professional GT2 (Nanoscribe GmbH & Co. KG) is utilised to 3D print the proposed microprobe. In consideration of the microprobe dimensions and the need for biocompatibility, a 25x objective and the corresponding resin, IP-S (Kramer et al. 2020), are chosen for fabrication. The “solid” is selected as the “fill mode” while the “slicing” and “hatching” distances are 1 μm and 0.5 μm, respectively. For the development of the printed sample, isopropyl alcohol (IPA) and propylene glycol methyl ether acetate (PGMEA) are used as solvents.

## Results and discussion

### Insertion

**Fig. 2:**
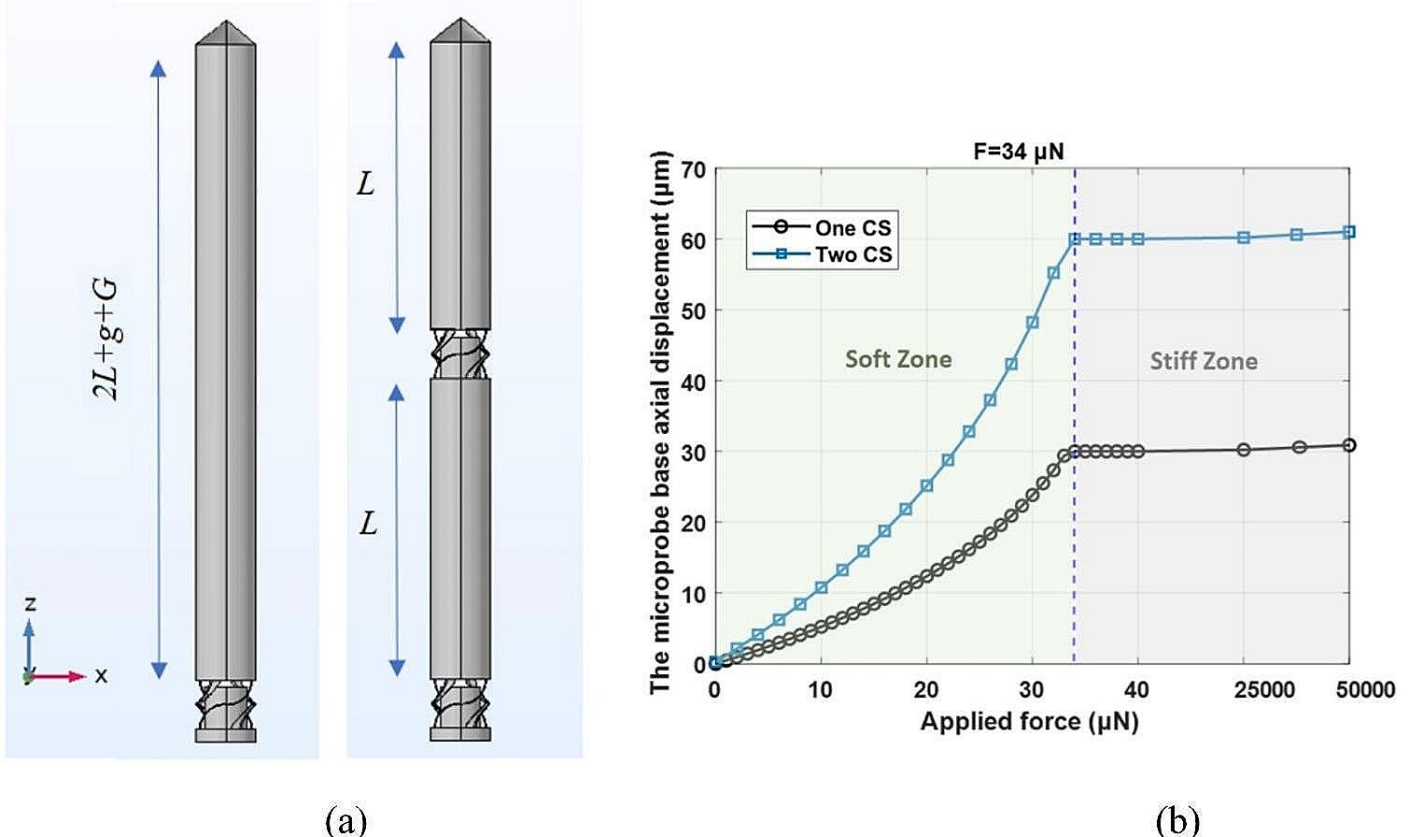
**(a)** The microprobes with one and two compressible structures (CS) and **(b)** the axial (longitudinal) displacements of the studied microprobes’ base against the applied force in the + z direction

The axial (longitudinal) displacements of the studied microprobes’ base (Fig. 2(a)) against different applied axial compressive forces, ranging from 0 to 50 mN, are obtained and compared in Fig. 2(b). As illustrated, by increasing the applied force from zero to 34 µN, there is an increment in both microprobes’ base displacement (in the + z direction). The microprobes are in their soft zone since the generated displacements are less than the corresponding gaps, *g* and 2*g*. The displacement of the proposed microprobe under a given force is always higher than that of the microprobe with one compressible structure (CS), which indicates a lower d*F*/d*w* and, therefore, a softer structure. *K*_*soft*_ =d*F*/d*w* for the proposed microprobe decreases from ≈ 1.09 to ≈ 0.29 N/m, indicating an equivalent stiffness of ≈ 25 kPa < *E*_*soft*_ < ≈ 93 kPa (refer to Eq. 2). The corresponding stiffness for the microprobe with one compressible structure is ≈ 30 kPa < *E*_*soft*_ < ≈ 150 kPa. By filling the gaps under *F* ≥ 34 µN, the proposed soft microprobe converts to a stiff one that may tolerate penetration force without any deflection (buckling). The proposed microprobe’s displacement for *F* = 50 mN is 61.031 μm which results in d*F*/d*w* ≈ 48,462 N/m and *E*_*stiff*_≈3.73 GPa (*η =* 1). Taking into account 4.2 GPa elastic modulus of the fabrication material, the correction factor can be calculated as *η ≈* 1.13.

In Table 2, a comparison of the transition time between stiff and soft modes is provided for some existing microprobes and the proposed microprobe.

**Table 2:** A comparison of some existing microprobes with the proposed microprobe

Proposed microprobe by	Stiff to soft transition time and type
(Wu et al. 2011)	30 min to 25 h - Irreversible
(Ware et al. 2012)	24 h - Irreversible
(Hess-Dunning and Tyler 2018)	few seconds - Irreversible
(Pas et al. 2018)	30 s - Irreversible
(Wen et al. 2019)	few minutes - Irreversible
(Zhang et al. 2020)	50 s - Irreversible
Current study	Immediately - Reversible

**Fig. 3:**
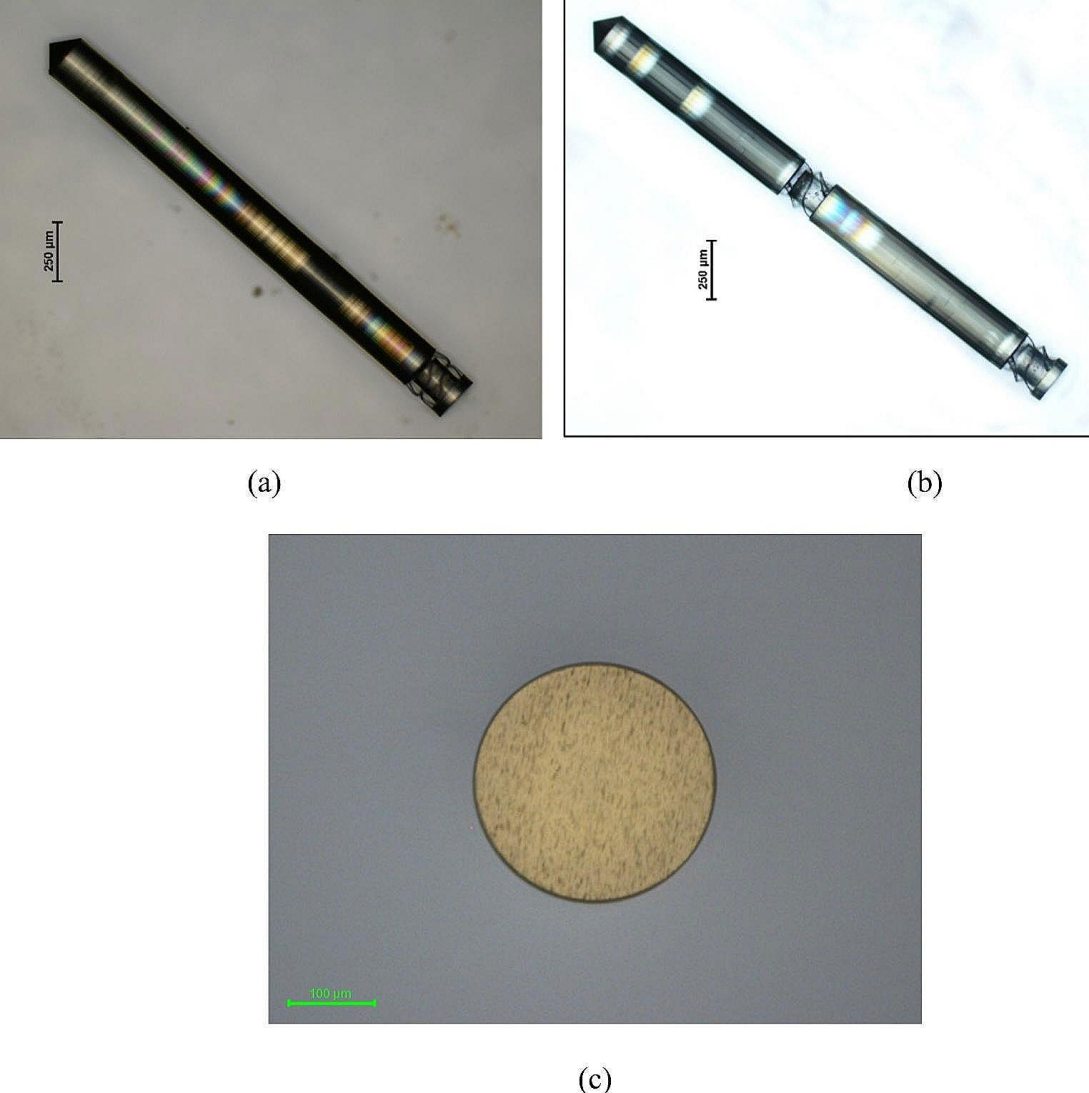
The 3D printed microprobes with **(a)** one and **(b)** two compressible structures on the ITO glass. **(c)** The cross-sectional view of the fabricated microprobe

Figure 3(a) and (b) illustrate the 3D printed microprobes with one and two compressible structures on an indium tin oxide-coated (ITO) glass, respectively. A cross-sectional view of the microprobe is depicted in Fig. 3(c). Although adding a third compressible structure may provide a softer structure theoretically, it causes the microprobe to be broken in the fabrication process.

**Fig. 4:**
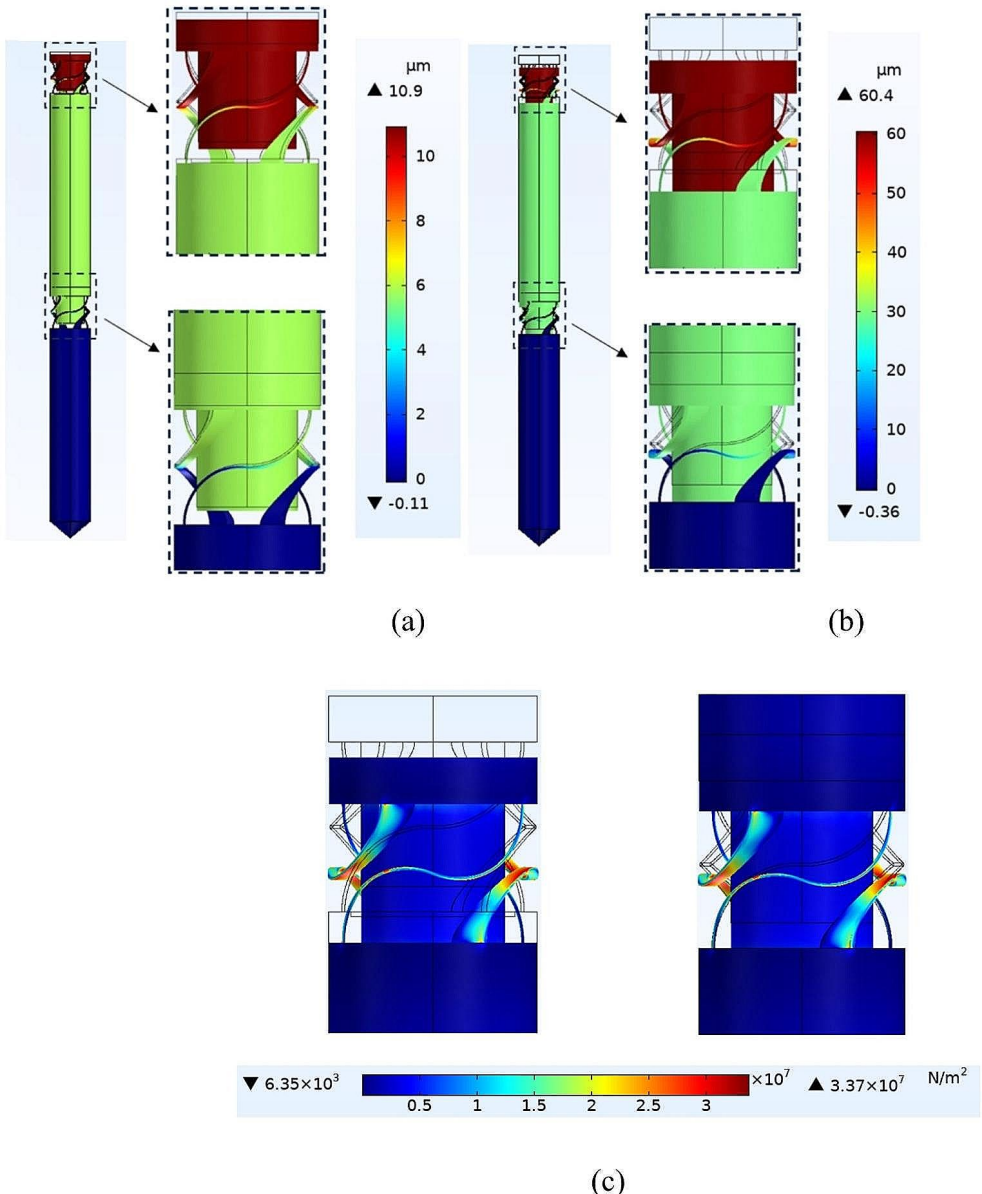
The longitudinal displacements of the proposed microprobe for **(a)***F* = 10 µN, and **(b)***F* = 100 µN. **(c)** The Von Mises stress distribution at the compressible structures of the proposed microprobe under *F* = 50 mN

The longitudinal displacements of the proposed microprobe and corresponding compressible structures are depicted under *F* = 10 µN and *F* = 100 µN in Fig. 4(a) and (b), respectively. As shown, the maximum displacements, which occur at the base, are 10.9 μm and 60.4 μm. Accordingly, the microprobe acts as a soft structure under *F* = 10 µN, whereas with *F* = 100 µN, the gaps are filled, and the structure becomes stiff. Figure 4(c) shows the Von Mises stress distribution at the two compressible structures of the proposed microprobe under *F* = 50 mN. The middle of the curved legs and the corresponding sharp corners experience the maximum stress.

**Fig. 5:**
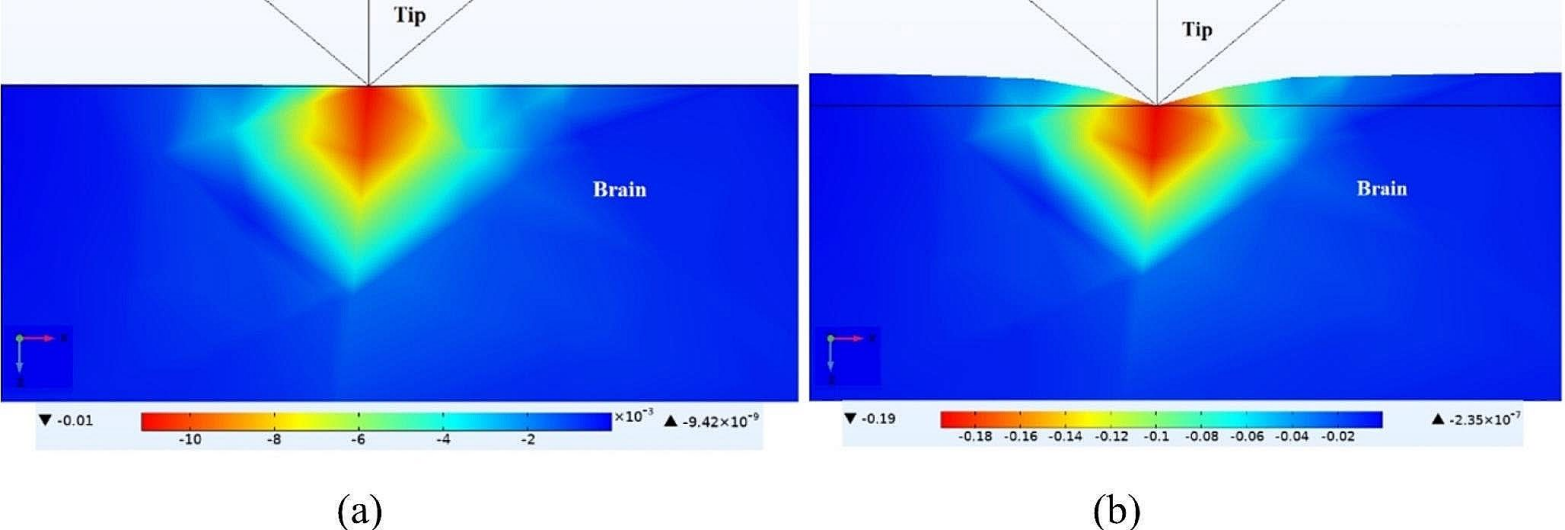
The maximum principal strain generated on the brain surface when the microprobe is pushed down **(a)** 0.1 μm and **(b)** 2 μm during insertion

After filling the gaps, the further force applied by the inserter is completely transferred to the brain surface, which causes a dimpling to appear on the surface just before insertion occurs. Figure 5(a) and (b) show the maximum principal strain generated on the brain surface when the microprobe is pushed down 0.1 μm and 2 μm during insertion, respectively. As illustrated, the dimpling results in a maximum strain of 0.19 exactly under the sharp tip. Although the strain causes acute tissue damage during insertion, chronic tissue damage during operation is the main parameter that determines a microprobe’s longevity (Biran et al. 2005).

### Operation

Applying a *W*=-30 μm brain longitudinal displacement generates ≈-22.5 μm and ≈-25.5 μm longitudinal displacements at the tip of microprobes with one and two compressible structures, respectively. As the created displacements are less than the corresponding gaps, the microprobes are in their soft zone and behave like a spring. The axial stiffness of the microprobes with one and two compressible structures are *K*_*soft*_ =0.53 N/m and *K*_*soft*_ =0.26 N/m, which lead to *E*_*soft*_ ≈ 40 kPa and *E*_*soft*_ ≈ 23 kPa, respectively.

**Fig. 6:**
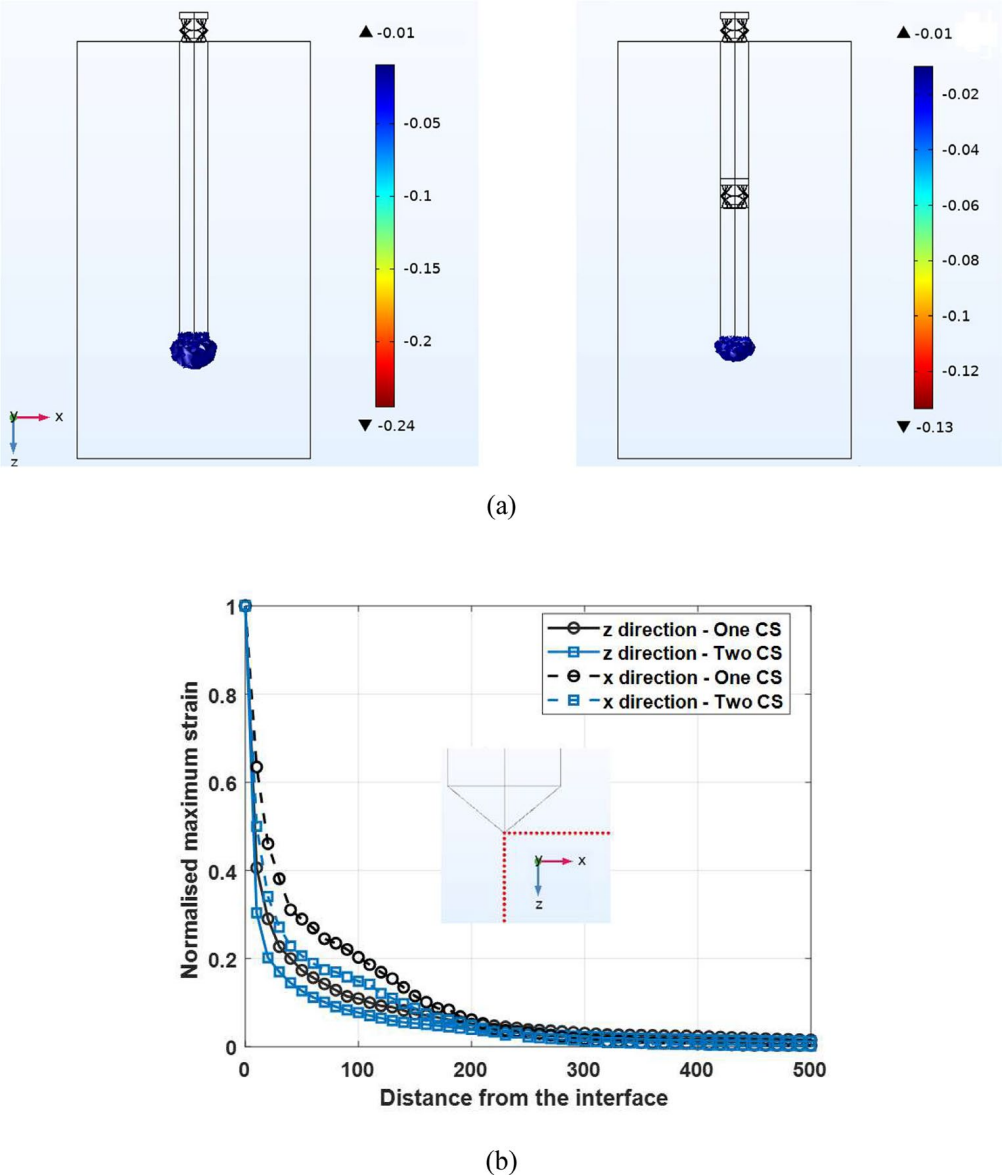
**(a)** The distribution of maximum principal strain with an absolute magnitude more than 0.01 on the surrounding neural tissue of the microprobes with one (left) and two (right) compressible structures under the brain longitudinal motion, *W*=-30 μm. **(b)** The normalised maximum strain against the distance from the interface of the neural tissue and the microprobes’ tip in the z and x directions

Figure 6(a) illustrates the maximum principal strain with an absolute magnitude of more than 0.01 generated around the studied microprobes under the brain longitudinal motion, *W*=-30 μm. As shown, consistent with the results reported by Subbaroyan et al. (Subbaroyan et al. 2005), only the tissue around the tip of the microprobes is affected and strain in the other areas is less than 0.01 (colourless areas). Furthermore, the strain generated around the proposed microprobe’s tip (right Figure) is ≈ 46% lower than that of the microprobe with one compressible structure (left Figure). The volume of the affected tissue is also smaller which leads to less tissue damage around the proposed microprobe. The generated maximum principal strain is normalised with respect to its value at the interface of the neural tissue and the studied microprobes’ tip. Figure 6(b) shows the normalised maximum strain against the distance from the interface of the neural tissue and the tip in the z and x directions (red points in the embedded Figure) for the studied microprobes. As depicted, the strain decreases exponentially as a function of the distance for both microprobes and approximately reaches zero at ≈ 300 μm distance from the tip.

**Fig. 7:**
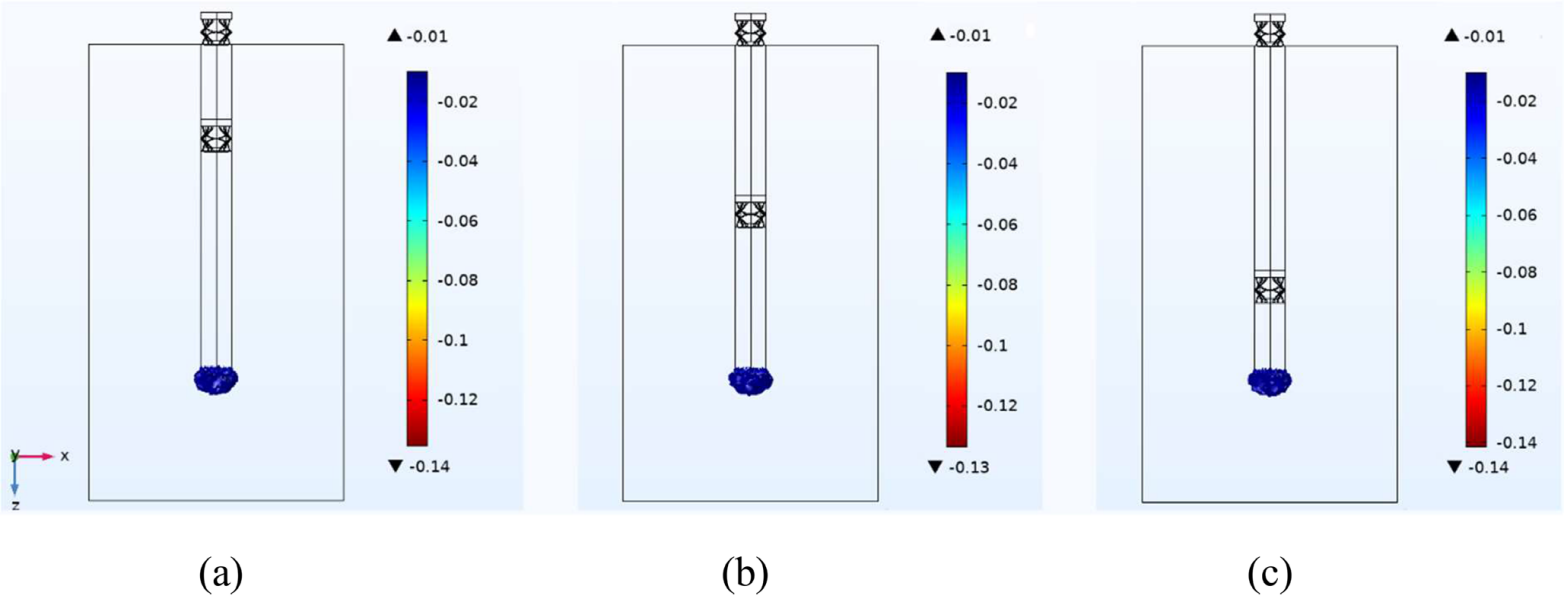
**(a)** The generated maximum principal strain by the proposed microprobe under the brain longitudinal motion, *W*=-30 μm, for different length ratios between the upper and lower cylinders, **(a)** 0.5, **(b)** 1, and **(c)** 2

Figure 7 illustrates the maximum principal strain generated by the proposed microprobe under the brain longitudinal motion, *W*=-30 μm, for different length ratios between the upper and lower cylinders. As depicted, the position of the second compressible structure has a negligible effect on the generated strain and the strain is minimum for the unit length ratio (Fig. 7(b)).

**Fig. 8:**
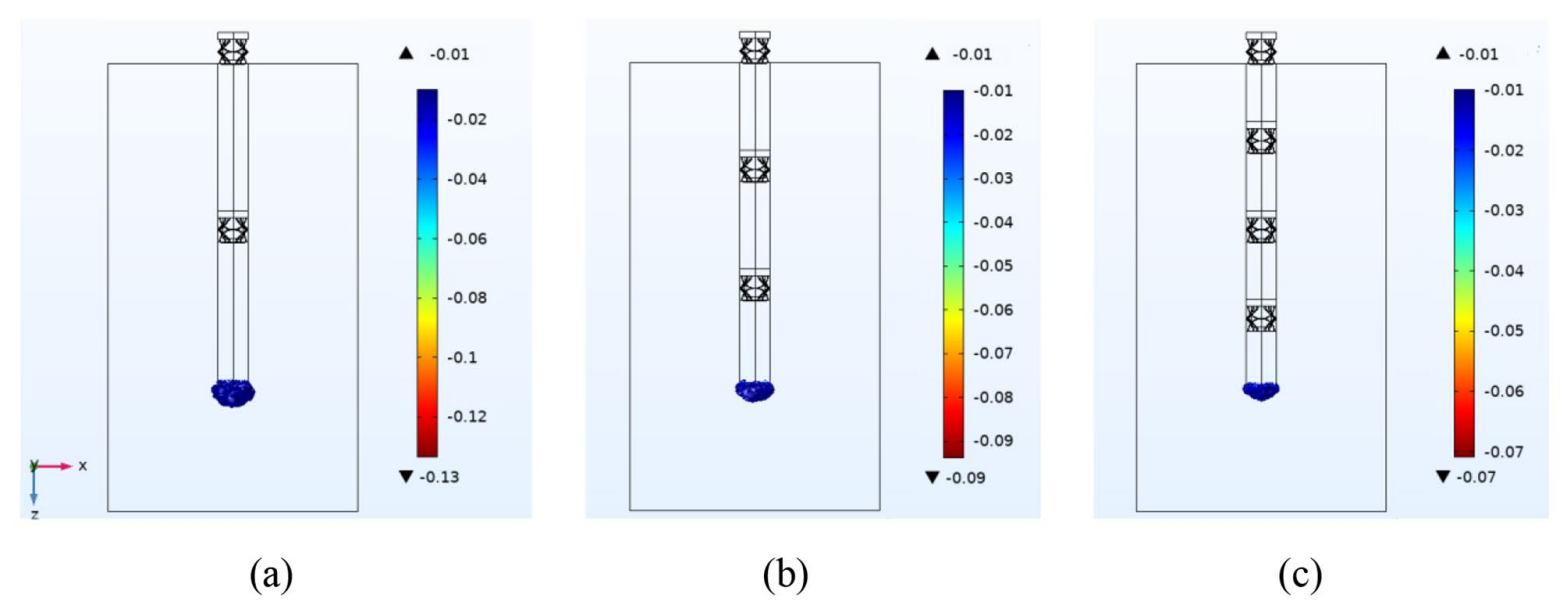
**(a)** The generated maximum principal strain under the brain longitudinal motion, *W*=-30 μm, by the microprobes with different numbers of compressible structures, **(a)** two, **(b)** three, and **(c)** four

Figure 8 compares the maximum principal strain generated under the brain longitudinal motion, *W*=-30 μm, by the microprobes with two, three, and four compressible structures. As shown, increasing the number of compressible structures enhances the microprobe’s flexibility, resulting in a lower strain. Nevertheless, the generated flexibility leads to fabrication failures for the microprobes with three and four compressible structures.

**Fig. 9:**
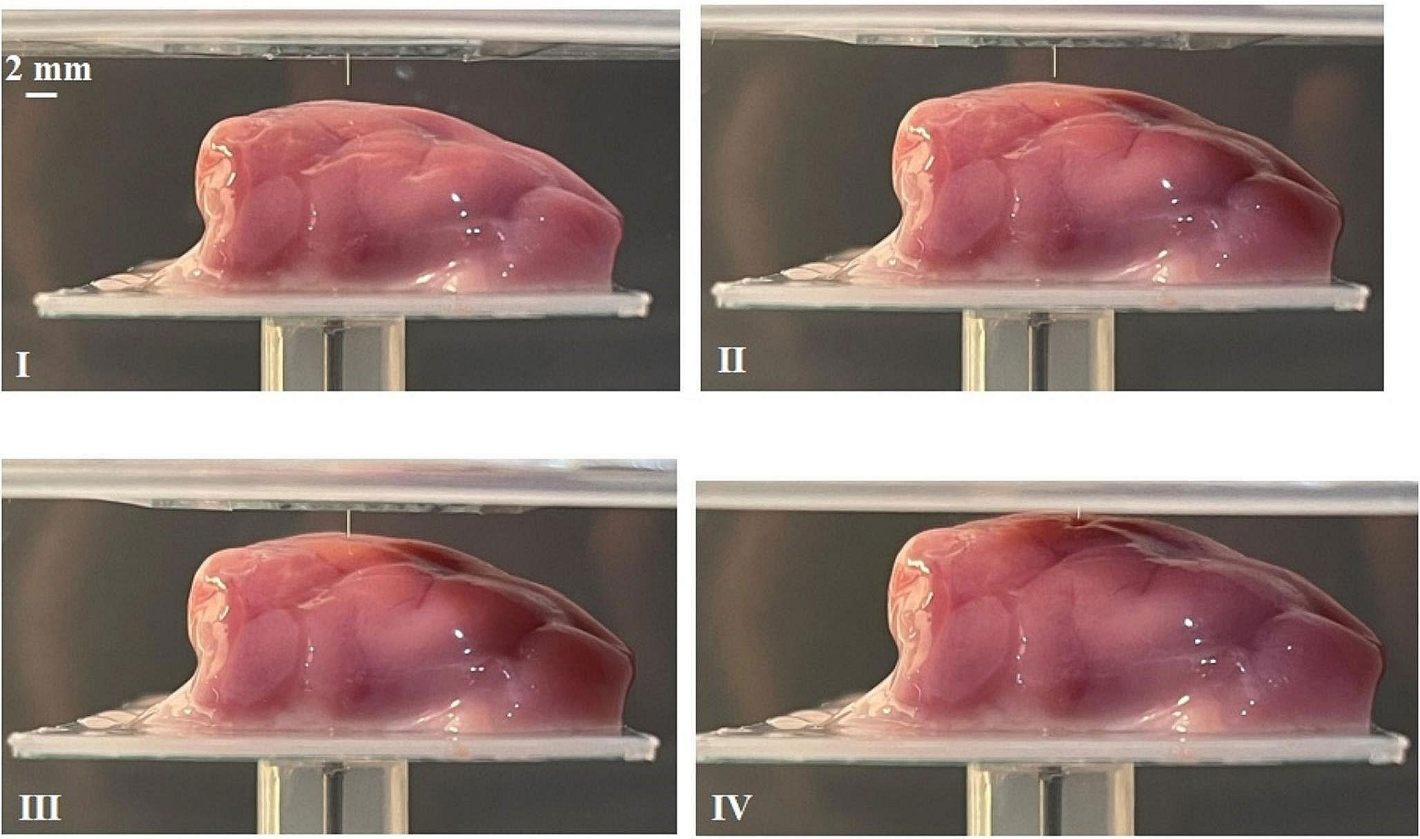
The insertion of the microprobe with two compressible structures into the lamb’s brain surface

**Fig. 10:**
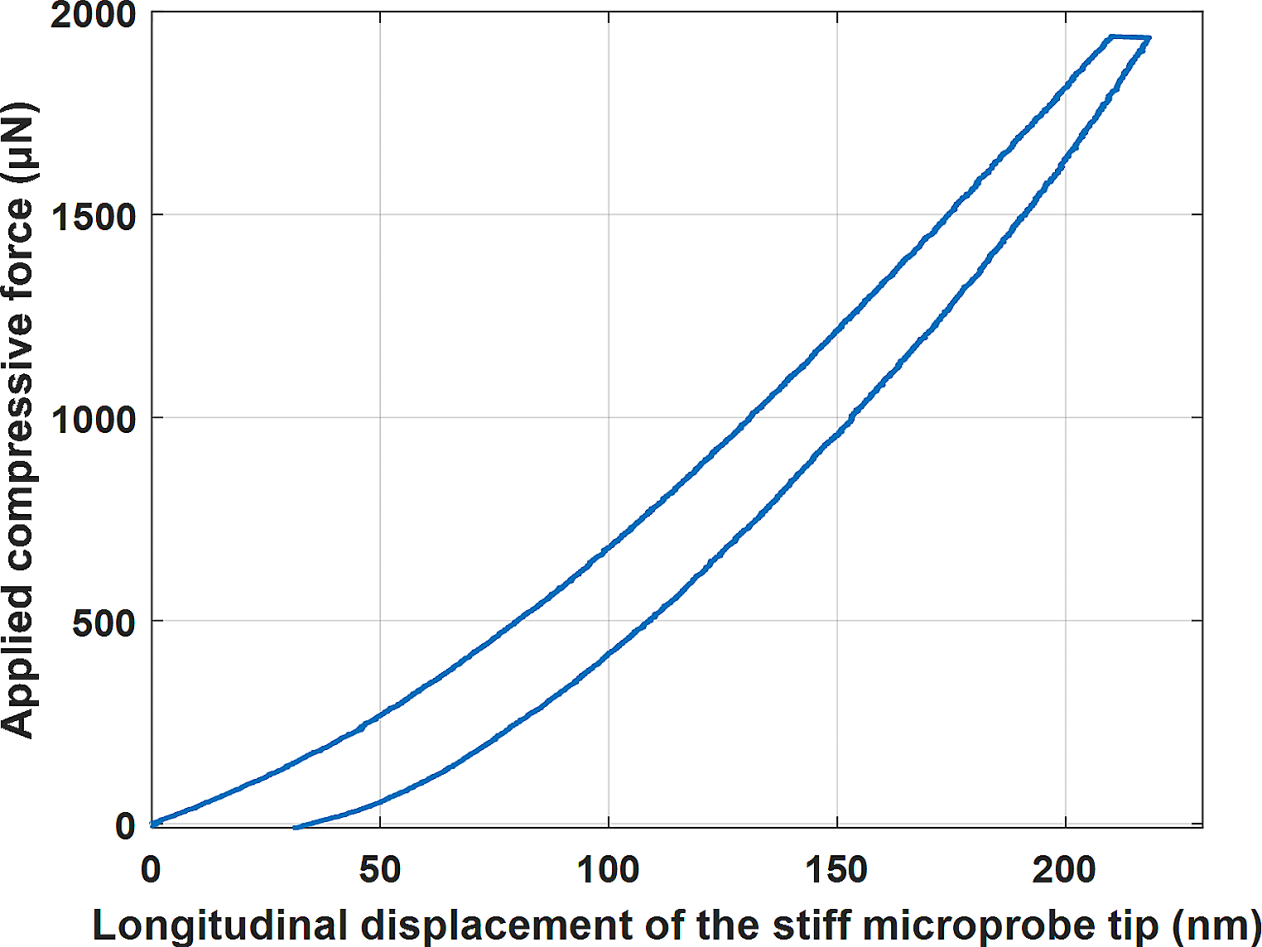
The applied compressive force by the Hysitron TI 950 Triboindenter against the longitudinal displacement of the stiff microprobe’s tip

To show that the microprobe could withstand the penetration force, a manual inserter is used to push the microprobe toward the surface of a lamb’s brain. Figure 9 illustrates how the microprobe is inserted without buckling into the brain. As shown, the microprobe, vertically attached to the substrate, approaches the brain surface (Fig. 9 (I-III)) until it finally penetrates the surface (Fig. 9 (IV)). Moreover, using a Hysitron TI 950 Triboindenter (Bruker), a compressive force of ≈ 1935 µN is applied to the proposed microprobe in the stiff zone to evaluate its resistance against buckling. The longitudinal displacement of the stiff microprobe’s tip against the applied force is depicted in Fig. 10. As illustrated, in the first 25 s, the applied force increases from zero to 1935 µN, resulting in the ≈ 218 nm longitudinal displacement. After keeping the applied force constant for 10 s, the force decreases to zero in 20 s, which results in the tip displacement decreasing to zero as well, confirming that there is no buckling.

## Conclusion

This study proposed a self-stiffening compliant intracortical microprobe which consists of two cylinders mounted on two identical compressible structures. The microprobe is stiff enough to withstand the insertion force while having an elastic modulus comparable to that of the brain during operation. As the soft microprobe becomes stiff once an axial force is applied by an inserter, it can switch between soft and stiff modes as many times as necessary to ensure accurate positioning with minimal tissue damage. The proposed microprobe’s behaviour was investigated during insertion and operation based on the finite element method. The equivalent elastic moduli of the microprobe during insertion and operation are ≈ 4.2 GPa and ≈ 23 kPa, leading to high resistance against buckling and great compliance with surrounding neural tissue, respectively. With two compressible structures, the maximum strain generated around the proposed microprobe under brain longitudinal motion is ≈ 46% lower than that of the microprobe with one compressible structure, which confirms the efficiency of the optimised design. There was no access to animal models to provide in-vivo test results and validate the microprobe during operation. Two-photon polymerisation technology was applied to 3D print the proposed microprobe, which was experimentally validated and successfully inserted into a lamb’s brain without buckling.

## Data Availability

The data that support the findings of this study are available upon reasonable request from the authors.
